# Conservation agriculture’s impact on total and labile organic carbon pools in calcareous and non-calcareous floodplain soils under a sub-tropical rice-based system

**DOI:** 10.1371/journal.pone.0293257

**Published:** 2023-11-08

**Authors:** Rakhi Rani Sarker, M. H. Rashid, Md. Ariful Islam, M. Jahiruddin, Khandakar Rafiq Islam, Mohammad Mofizur Rahman Jahangir

**Affiliations:** 1 Department of Soil Science, Bangladesh Agricultural University, Mymensingh, Bangladesh; 2 Soil Science Division, Bangladesh Institute of Nuclear Agriculture, Mymensingh, Bangladesh; 3 On-Farm Research Division, Bangladesh Agricultural Research Institute, Pabna, Bangladesh; 4 Soil, Water and Bioenergy Resources, The Ohio State University, Columbus, Ohio, United States of America; Central Research Institute for Dryland Agriculture, INDIA

## Abstract

To evaluate the effects of conservation agriculture (CA) on SOC pools and their lability, field experiments (2015–2020) were conducted on contrasting soils under subtropical climates. The experiment on non-calcareous soils, was comprised of tillage (minimum [MT] vs. conventional [CT]) in main plots, cropping systems (Wheat [*Triticum aestivum]*—Aus and Aman rice [*Oryza sativa* L.], WRR; Lentil [*Lens culinaris*]—Aus and Aman rice, LRR; and Mustard [*Brassica nigra*]- Boro and Aman rice, MRR) in the sub-plots, and crop residue (with or without 20% residue) in the sub-sub plots. The experiment on calcareous soils, was comprised of tillage (strip-till, ST; no-till, NT; and CT) and crop residue (high residue, HR at 50% by height vs. low residue, LR at 15%). Results showed that the MT had higher SOC contents by 18.8% than the CT in non-calcareous soils. Likewise, SOC was 12.5% and 6.7% higher in the NT and ST, respectively, than in the CT in calcareous soils. Significantly higher particulate organic (POC), permanganate oxidizable (POXC), and microbial biomass carbon (MBC) were observed in the MT, NT, and ST than in the CT at both locations. Reduced tillage with residue retention under LRR had a higher SOC, including labile C pools compared to WRR and MRR systems. Similarly, carbon management index (1.2–1.5 and 1.0–1.2) in both soils had significant positive correlations with SOC lability via POXC, POC, and MBC pools, indicating a SOC sequestration potential. In conclusion, our results showed positive effects of CA on SOC and its lability across soils.

## Introduction

Soil and crop management practices play an important role in soil biological properties associated with residue decomposition, organic carbon (SOC), nitrogen dynamics, and soil health. To overcome the challenges in SOC depletion associated with poor soil fertility, low crop productivity, and higher rates of greenhouse gas emissions, information on management practices that favor SOC accumulation is much needed. Any alteration in SOC contents may influence the microbial community and biological activities, N mineralization, greenhouse gas emissions, and SOC sequestration [1–4]. Conventional management practices, especially plowing (CT), are responsible for negatively affecting the agroecosystem services related to sustainable soil health, environmental quality, and crop productivity. Soil management that includes intensification and diversification of crops, reduced soil disturbance, and residue retention can increase SOC accumulation and improve the sustainability of agricultural systems. It is widely recognized that no-till (NT) practices increase soil aggregation, improve other soil properties, and favorably influence SOC accretion [5]. The distribution crop residue on the surface and subsurface soils changes dramatically with the initiation of NT practices to favor SOC accumulation. Crop residue management and no-till or shallow tillage play a significant role in protecting surface soil against soil erosion, improving biodiversity, and enhancing nutrient and fertilizer-use efficiency [6–8].

The SOC is recognized as one of the core indicators of soil health in the face of the challenges of food security and environmental compatibility to improve the sustainability of agroecosystems [9]. The prolongation of farmers’ practices of intensive tillage without residue retention has accelerated SOC decline over time. Higher amounts of crop residue return in strip tillage (ST) can be an effective strategy in slowing the loss of SOC and enhancing C sequestrations in the intensive rice-based production systems of the Eastern Indo-Gangetic Plain of Bangladesh [10]. SOC, especially labile C fractions, can be increased by crop residue management that is characterized by a rapid turnover, and thus, recommended as early and sensitive indicators of the effects of conservation management practices on soil fertility [11].

Conservation agriculture (CA) is an eco-friendly farming system composed of reduced soil disturbance, residue retention, and crop rotation, which has been reported to enhance soil health and reduce the cost of production [10, 12, 13]. Worldwide crop production area under CA is around 180 M ha, which was equal to 12.5% of the total global cropland in 2015–16 [14]. It aims to sustain crop production to improve agroecosystem services [15] and is considered by many as “farming for the future” [16, 17]. It helps increase SOC sequestration, improve biological activities, and reduce nutrient losses via erosion and as greenhouse gases. Soil biological activities are driven by organic carbon inputs from crop residue, cover crops, manure applications, or organic amendments, all of which have a strong positive effect on the composition, size, activity, and efficiency of the soil biological community [18]. Likewise, an understanding of soil chemical properties under different management practices and total soil N is required to better understand SOC dynamics. Studies on SOC sequestration and sustainable crop production are the primary focus in tropical regions, where the rate of SOC decomposition is high [5].

Soils can be sources or sinks of tropospheric CO_2_ depending on land use and management of agricultural practices. Maintenance of SOC is important for the enhancement of agricultural productivity and for reduction in CO_2_ emissions. SOC is not only an important source of C for soil processes, but also a sink for SOC sequestration [19]. While conventional agricultural practices can reduce SOC content, lead to soil deterioration, and reduce soil productivity, by contrast, the adaptation of a CA system can minimize the adverse effects of crop production via climate change mitigation and adaptation [10, 20]. By changing plowing to NT systems, or by improved management practices, SOC sequestration can be increased with a new equilibrium within several decades.

Reduced tillage or NT in conjunction with crop residue management may benefit nutrient-depleted intensive agroecosystems in southern and Southeast Asia. This is because the response of the changes in management practices, such as crop residue addition and improved nutrient management, are observed more rapidly and in greater magnitude in highly depleted soils in the tropical regions than in the temperate regions [21]. Limited information is documented on SOC sequestration and the labile SOC pools after the introduction of CA practices from the CT rice-based systems to NT system in South Asia [5], especially in Bangladesh. Globally, there are ample studies on SOC sequestration, but information on SOC pools and dynamics after conversion of CT to conservation tillage along with crop residue retention in rice-based systems is limited. In addition, it is still up for debate whether conservation agriculture increases SOC, has no effect on C accumulation, or has negative effects. We hypothesize that, as one the holistic management components, CA will influence SOC pools and the soil C management index (CMI) in rice-based sub-tropical agroecosystems. The objective of our study was to evaluate the impact of different tillage and residue managements on SOC pools, carbon lability, and CMIs in two contrasting floodplain soils (calcareous vs. non-calcareous) under sub-tropical, rice-based cropping systems.

## Materials and methods

### Experimental sites

Field experiments were conducted using a combination of tillage, cropping system, and residue management as independent factors at two different agroecological zones (AEZ 9 & AEZ 11) in Bangladesh [22]. Both experiments were established under sub-tropical monsoon climatic conditions in 2015.

The first experiment was conducted in Non-Calcareous Dark Grey Floodplain soils on tillage studies (CT vs. MT) at the research farm of the Bangladesh Agricultural University under AEZ 9 (at 24.54°N lat. and 90.50°E long. at 18 m above the mean sea level). The soil is a Sonatala silt loam series (Hyperthermic Aeric Haplaquepts) that contains 60, 30, and 10% sand, silt, and clay, respectively, and has a pH of 6.12. Total N (TN), available P, available S, and exchangeable K contents at 0 to 7.5 cm depth were 0.11%, 12.3 mg/kg, 14.1 mg/kg, and 31.2 mg/kg, respectively.

The other experiment was conducted in Calcareous Dark Grey Floodplain soils on CA studies (tillage practices: CT, ST, and NT; and residue levels: high residue– 50% rice residue and 100% legume residue; low residue– 15% rice residue and without any legume residue on the surface; cropping system: lentil—mungbean—monsoon transplanted Aman rice) at Pulses Research Centre of Bangladesh Agricultural Research Institute, Ishurdi, Pabna (24° 7′ 15.08″ N lat. and 89° 4′ 45.95″ E long. at the elevation of 26 m above the sea level) under High Ganges River Floodplain (AEZ 11). The area is a medium highland with medium to good drainage under sub-tropical monsoon climatic conditions. The soil is a Sara loam series (Aquic Eutrochrepts) and contains 38, 40, and 22% sand, silt, and clay, respectively. It has a pH of 7.93 with TN, available P, available S, and exchangeable K contents of 0.12%, 10.9 mg/kg, 12.2 mg/kg, and 97.5 mg/kg, respectively.

### Experimental treatments and cultural practices

The first experiment was established in a split-split plot with three replications at the non-calcareous site with Factor 1—tillage (minimum tillage, MT vs. conventional tillage, CT); Factor 2—combined with three cropping systems of Wheat—T. Aus—T. Aman rice (WRR), Lentil—T. Aus—T. Aman rice, (LRR), and Mustard–Boro—T. Aman rice (MRR); and Factor 3—two crop residue management practices (20% [HR] vs. no previous residue [NR]). Tillage systems were arranged in the main plots, cropping systems were in sub-plots, and residue retention was in sub-sub-plots. Nine crops in three phases were accommodated annually. While in MT, plowing was performed at 0–5 cm depth with one passing of a rotary tiller. In CT, plowing was performed at 0–15 cm depth with four passes of a rotary tiller.

While the MRR system (mustard, Boro, and Aman rice) was grown from November to January, February to May, and July to November, the WRR system (wheat, Aus, and Aman rice) was grown from November to March, April to July, and July to November. Similarly, the LRR system (lentil, Aus, and Aman rice) was grown from November to March, April to July, and July to November. Agronomic cultural practices were performed from time-to-time including weeding, gap filling, irrigation, and spraying of herbicides and insecticides as required.

Each replicated plot was cultivated three times annually for the three-crop phases, as per treatment. During final land preparation, the total amount of P, K, S, and Zn was applied in the form of triple superphosphate, muriate of potash, gypsum, and ZnSO_4_.7H_2_O, respectively. For minimizing the N loss and to increase N-use efficiency, the urea was applied in three equal splits for rice and wheat, and two equal splits for lentil. One-third of the urea fertilizer was applied to the individual plots at 10 d after root establishment, the second split was applied at 25 d, and third split was applied at 45 d after transplanting. Each crop was fertilized as per the recommendation of the Fertilizer Recommendation Guide [23].

In contrast, the second experiment was a two-factorial split plot experiment consisting of four replications at the calcareous site. Treatments were tillage (strip tillage [ST], no-till [NT], and conventional tillage [CT]) combined with two previous crop residues (high at 50% rice residue + 100% legume residue [HR]) vs low at 15% rice residue + 0% legume residue [LR]) in a Lentil—Mungbean—T. aman (LMR) cropping system. In CT, plowing was performed at 0–15 cm depth with four passes of a rotary tiller. For strip, a Versatile Multi-Crop Planter was used for seed and fertilizer placement in 3 cm wide furrows with 20 cm (wheat and rice) and 30 cm (mustard and mungbean) wide undisturbed soils between with crop residue retained in the inter-row.

### Soil sampling and processing

Soil core samples (1.95 cm internal dia.) were collected randomly after harvesting of the T. Aman rice using an auger at 0–7.5 and 7.5–15 cm depths and mixed to obtain a composite sample for each replicated plot. The fresh soil samples were temporarily stored in a refrigerator at 4°C. A portion of soil sample was air-dried, ground with a porcelain mortar and pestle, and sieved through a 2-mm sieve prior to physical and chemical analysis.

### Soil microbial biomass and associated biological properties

Soil microbial biomass carbon (MBC) was determined by the chloroform fumigation-extraction method [24]. A 10-g sample of field-moist soil (adjusted at 70% moisture content) was fumigated with alcohol-free chloroform in a vacuum desiccator for a period of 72 h in a dark at room temperature (~ 25°C). After removing the chloroform by vacuum extraction, soils were extracted with neutral 0.5-M K_2_SO_4_ solution at 100 rpm for 45 min on a horizontal shaker. Non-fumigated soils were similarly extracted and used as a control. All soil extracts were filtered through Whatman 42 paper and analyzed for extracted carbon using the acid dichromate wet oxidation method. The MBC was calculated as follows:

MBC=Fc×kc (Eq 1)

Where Fc represents the flush of C from the difference between (C extracted from the fumigated soil–C extracted from the non-fumigated soil), and the Kc is the extraction factor (0.45).

Soil basal respiration (BR), as a measure of antecedent biological activity, was determined by trapping CO_2_ with 5-mL 0.1 M NaOH using the acid-base titration method [25]. In brief, a 20-g field-moist soil sample was placed in a mason jar (closed chambers) in the presence of plastic vials containing 10-ml 0.1M NaOH and water, and incubated at 25°C in the dark for a period of 10 d. The amount of CO_2_-C trapped in the alkali was determined by titration with 0.1 M HCl to a phenolphthalein endpoint. A vial without any soil was incubated as a control.

### Soil total organic, permanganate oxidizable, and particulate organic carbon pools

Soil organic carbon was determined on finely-ground (< 125-μm) soil by following the Walkley-Black wet oxidation method [26]. Particulate organic carbon (POC) was extracted using the dispersion method [27]. Briefly, a 20-g sample of soil (<2.00-mm) was mixed with 100 ml of 10% sodium hexametaphosphate solution followed by shaking in a horizontal shaker at the rate of 130 rpm for a period of 18 h. The material retained on the sieve, defined as total particulate organic matter (> 53 μm), was dried at 50°C for 12 h. After shaking, the dispersed soil suspension was passed through a 53-μm sieve and washed with a weak jet of distilled water. The sand and organic residues retained on the sieve as particulate organic matter (> 53 μm) were air-dried at 50°C for 12 h. After drying, the sand and particulate organic matter were ground in a porcelain mortar and pestle, passed completely through a 0.149 mm sieve. Then sieved samples were weighed and analyzed for their C contents, representing the POC. Soil active carbon or permanganate oxidizable carbon (POXC) was determined using the mild KMnO_4_ oxidation method [28]. Briefly, 5 g of air-dried soil in a 50-mL plastic centrifuze tube was reacted with 20 ml of 0.02-M KMnO_4_ (pH 7) followed by shaking the suspension at 2,000 rpm for 2 min. After shaking, the suspensions were centrifuged at 3,000 rpm for 5 min to obtain soil-free aliquot. The absorbance of the diluted solution was measured spectrophotometrically at 565 nm using a series of standard curve solutions of 0.005, 0.01, 0.0125, 0.025, 0.05, and 0.1-M.

### Soil organic carbon lability and management indices

Soil carbon management index (CMI) was calculated by using the measured SOC, MBC, POC, and POXC data [29] as follows:

CMI=CPI×CLI (Eq 2)

Where CPI is the C pool index and CLI is the C lability indexes, which were calculated as:

CPI=SOCinthetreatmentsoil/SOCinthecontrolsoilunderCT

CLI=CLinthetreatmentsoil/CLinthecontrolsoilunderCT

Where CL refers to the lability of C (CL = Labile C / Non-labile C).

The POXC pool consisted of the labile C pool, which was measured as a percentage of SOC. The non-labile C pool was estimated by subtracting the POXC content from the SOC. The calculated CMIs were normalized (CMI) by dividing the values with the highest CMI values in the database to a relative scale of > 0 to ≤ 100. Higher CMI values are considered better indicators of SOC accumulation and lability in response to management treatments.

#### Soil organic carbon stocks

The SOC, POC, POXC, and MBC stocks, at both 0–7.5 and 0–15 cm depths, were calculated by multiplying their concentrations (g/kg or mg/kg) with respective sample depth (m) and antecedent bulk density (ρb as Mg/m^3^) values [30].


Carbonstock(Mg/haorkg/ha)=(ρb*depth*SOC*10000) (Eq 3)

### Soil basic properties

Soil pH was determined by a glass electrode pH meter using a soil: water ratio of 1:2.5 [31]. The standard core method was followed to determine soil bulk density. Total N content in soil was determined by the Kjeldahl method [26]. Exchangeable K content of soil was determined by extraction with 1M NH_4_OAc, pH 7.0 solution followed by flame photometer. Available S content was determined by extracting soil sample with CaCl_2_ (0.15%) solution [26].

### Statistical analysis

For the first experiment, a split–split plot three-way analysis of variance (ANOVA) was performed using tillage, cropping pattern, and residues as fixed variables. For the second experiment, a split-plot two-way analysis of variance was performed using tillage and residues as fixed variables. Data were statistically analyzed to ascertain the significant differences for main effects and interactions on dependent variables in response to the impact of independent variables. If there was a statistically significant interaction, then the interaction was presented, but the main effects of the treatment factors that were involved in this interaction were not reported. Otherwise, only the main effects of treatments were presented. All statistical analyses were considered significant at p ≤ 0.05, unless otherwise mentioned. All the statistical analyses were performed on Statistics 10.0 Package.

## Results

### Management systems impact on microbial biomass and associated biological properties

**Fig 1:**
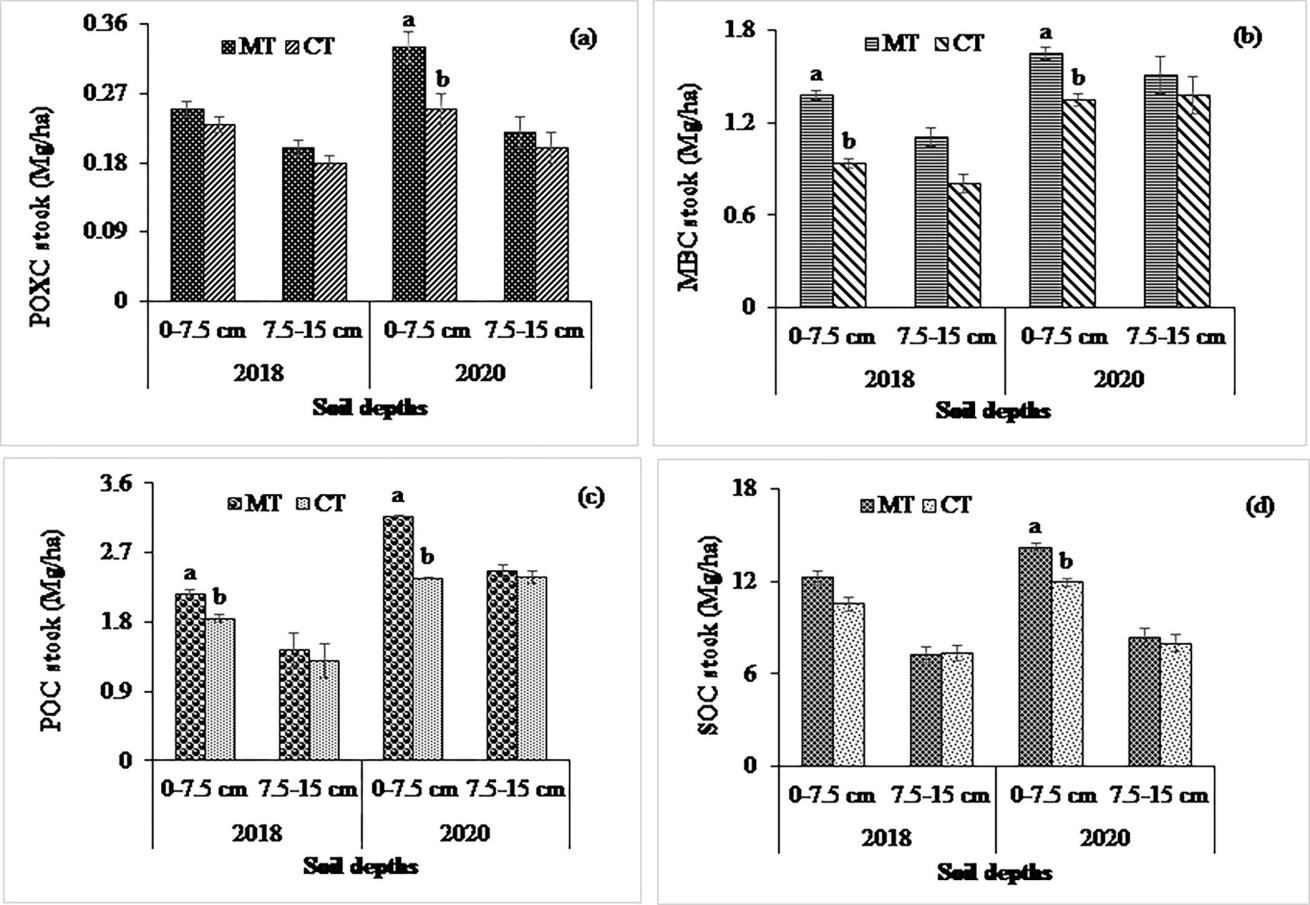
Impact of tillage system on (a) POXC, (b) MBC, (c) POC, and (d) SOC stocks at 0–7.5 and 7.5–15 cm soil depths in 2018 and 2020 at non-calcareous site. Data reported are means ± standard errors.

**Fig 2:**
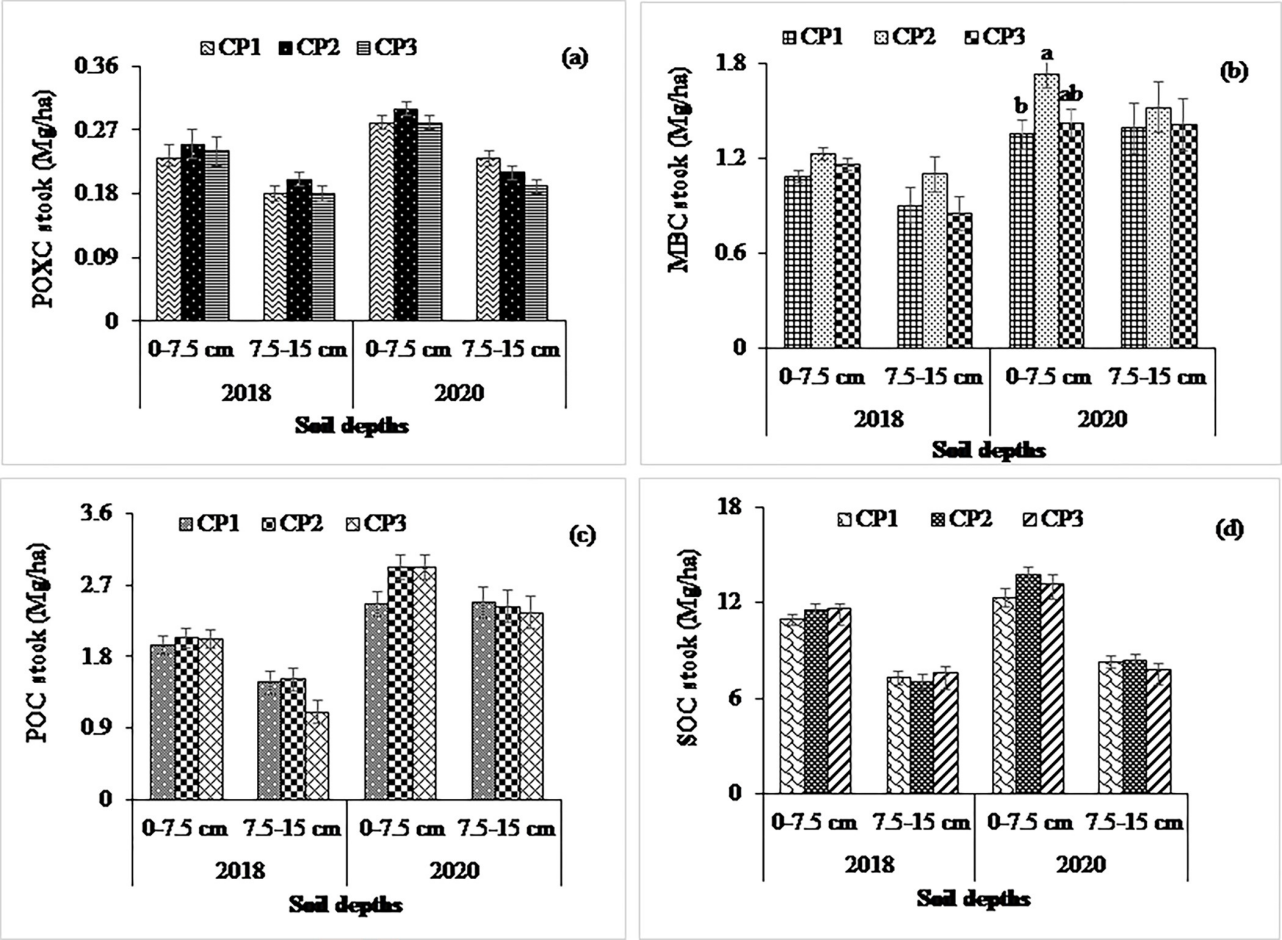
Impact of cropping pattern on (a) POXC, (b) MBC, (c) POC, and (d) SOC stocks at 0–7.5- and 7.5–15 cm depths in 2018 and 2020 at non-calcareous site. Data reported are means ± standard errors.

**Fig 3:**
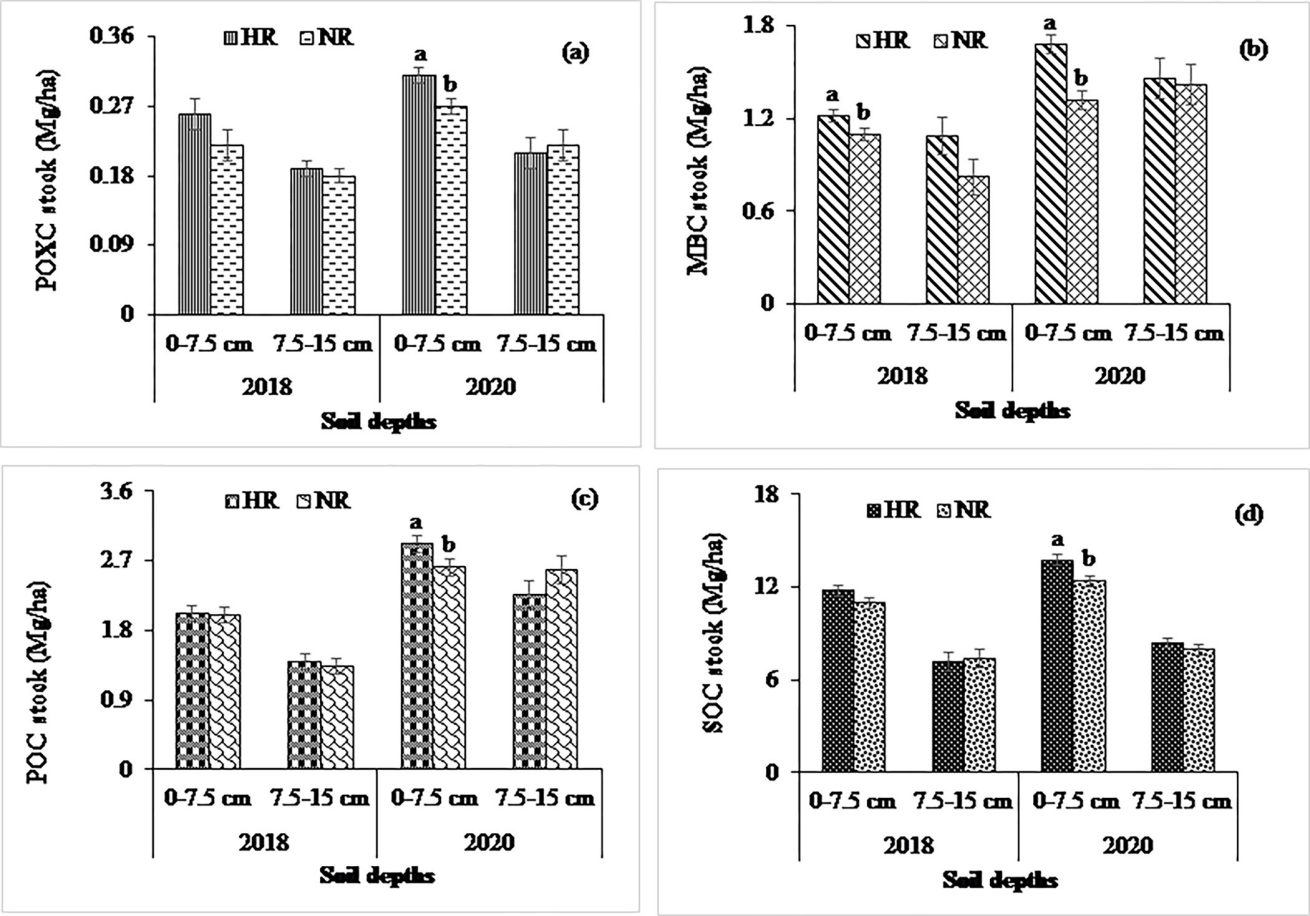
Impact of residue retention on (a) POXC, (b) MBC, (c) POC, and (d) SOC stocks at 0–7.5- and 7.5–15 cm depth in 2018 and 2020 at non-calcareous site. Data reported are means ± standard errors.

**Fig 4:**
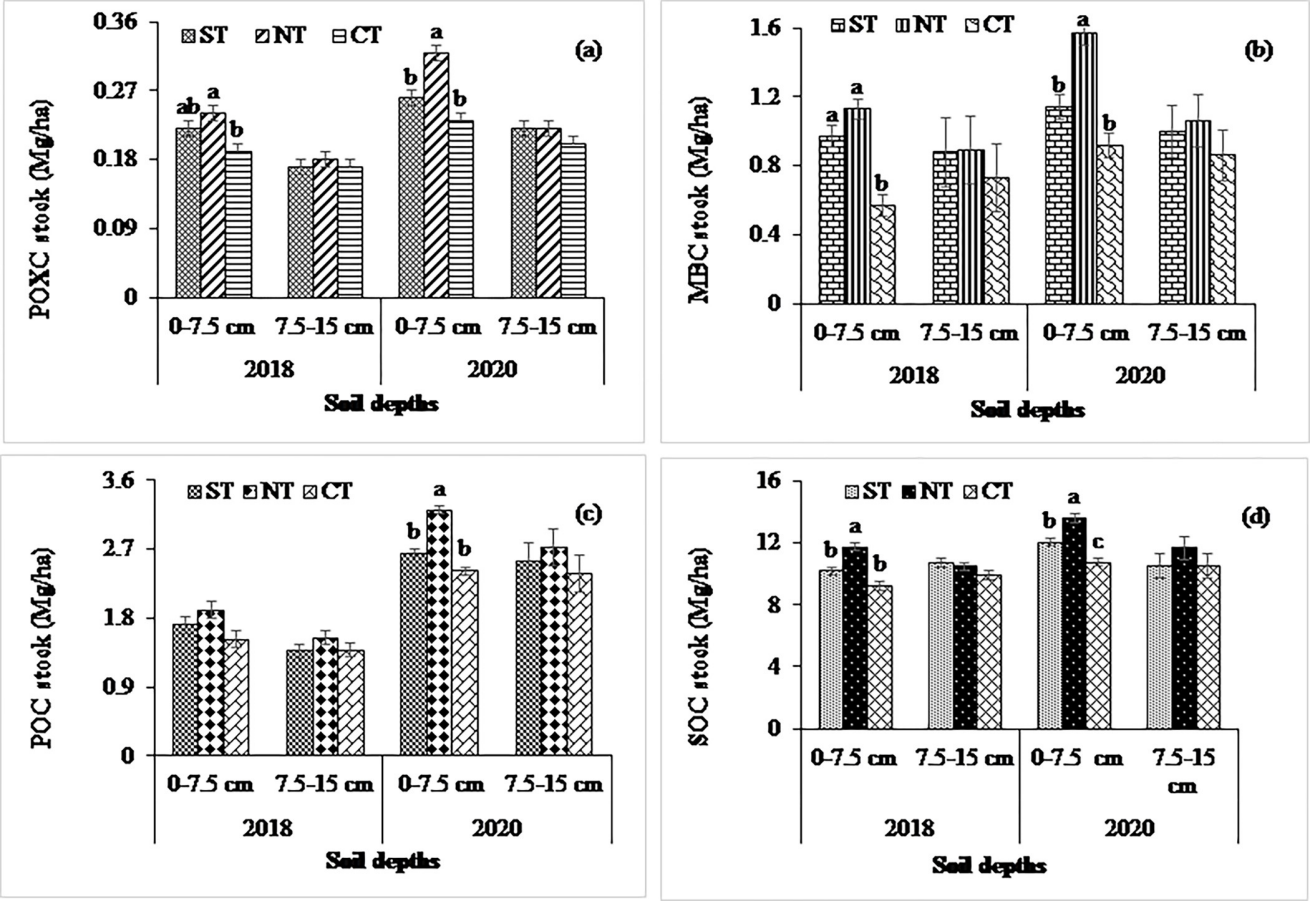
Impact of tillage system on (a) POXC, (b) MBC, (c) POC, and (d) SOC stocks at 0–7.5- and 7.5–15 cm soil depths in 2018 and 2020 at calcareous site. Data reported are means ± standard errors.

**Fig 5:**
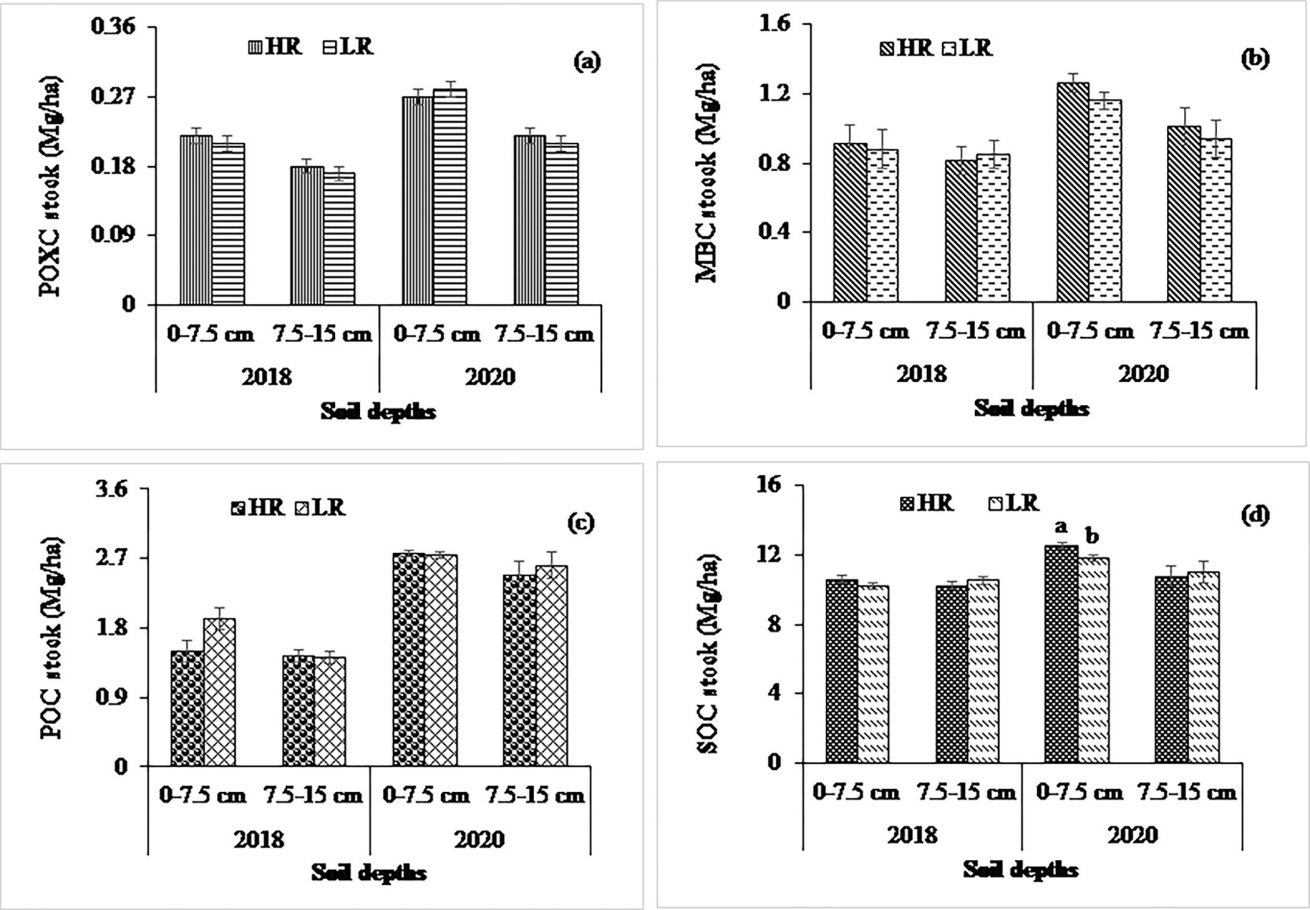
Impact of residue retention on (a) POXC, (b) MBC, (c) POC, and (d) SOC stocks at 0–7.5- and 7.5–15 cm soil depths in 2018 and 2020 at calcareous site. Data reported are means ± standard errors.

At the non-calcareous site, experimental treatments significantly influenced the MBC stock in both years except the effect of cropping system at 0–7.5 cm in the year 2018 (Figs 1B, 2B and 3B). The higher MBC stock was observed in the MT (1.38 and 1.65 Mg ha^-1^ in 2018 and 2020, respectively, at 0–7.5 cm soil depth) treatment than in the CT (0.94 and 1.35 Mg ha^-1^ in 2018 and 2020, respectively, at 0–7.5 cm soil depth), which was statistically significant in both years. In contrast, the MBC stocks at the calcareous site were significantly influenced by tillage treatments at 0–7.5 cm depth in both years, whereas the higher values were observed in the NT compared to the ST and CT in both years. Considering crop residue management, MBC were statistically similar, being higher in the HR than in the LR treatments at both depths (Figs 4B and 5B).

The ANOVA indicated that tillage had significant effects on BR rates at 0–7.5 cm depth in both years at the non-calcareous site. Average BR rates were significantly higher in the CT (30.60 mg/kg/d) than in the MT (27.90 mg/kg/d) systems in both 2018 and 2020, showing mean values of 29.60 mg/kg in the MT and 34.70 mg/kg in the CT. With regard to the cropping system, all values were similar and nonsignificant except at the 7.5–15 cm depth in 2018. BR was statistically significant in crop residue retention except at 7.5–15 cm depth in 2020 (Table 1). At the calcareous site, in contrast, tillage had significant effects on BR at 0–7.5 cm depth in both years. Average BR was highest in the CT than in the ST and NT in both cases. The average BR rates were 28.95, 36.90, and 39.97 mg/kg/d in the NT, ST, and CT systems, respectively, at 0–7.5 cm in 2018 and 31.45, 38.65, and 43.7 mg/kg/d in the NT, ST, and CT systems, respectively, in 2020. With regard to residue retention, the BR rates at HR and LR were similar and non-significant in both years (Table 2).

**Table 1:** Effects of management practices on soil organic carbon (SOC), basal respiration (BR), permanganate oxidizable carbon (POXC), and particulate organic carbon (POC) contents at non-calcareous site.

Factors	SOC (%)				BR (mg CO_2_/kg/d)				POXC (mg/kg)				POC (g/kg)			
	2018		2020		2018		2020		2018		2020		2018		2020	
**Tillage system**	**0–7.5 cm**	**7.5–15 cm**	**0–7.5 cm**	**7.5–15 cm**	**0–7.5 cm**	**7.5–15 cm**	**0–7.5 cm**	**7.5–15 cm**	**0–7.5 cm**	**7.5–15 cm**	**0–7.5 cm**	**7.5–15 cm**	**0–7.5 cm**	**7.5–15 cm**	**0–7.5 cm**	**7.5–15 cm**
MT	1.32a	0.69	1.52a	0.78	27.90b	19.20	29.60b	22.20	270	194	353a	216	2.36a	1.38	3.37a	2.32
CT	1.13b	0.73	1.28b	0.76	30.60a	20.60	34.70a	24.50	255	183	277b	195	1.99b	1.28	2.55b	2.28
SE	0.02	0.06	0.02	0.03	0.61	0.57	0.92	0.47	6.68	8.31	10.90	14.62	0.03	0.23	0.09	0.14
Level of sig.	*	ns	*	ns	*	ns	*	ns	ns	ns	*	ns	*	ns	*	ns
**Cropping system**																
CP1	1.19	0.71	1.33b	0.78	30.20	20.30ab	32.00	25.20	253	182	311	226	2.13	1.45	2.66b	2.34
CP2	1.26	0.68	1.47a	0.79	27.60	17.40b	32.50	22.30	272	201	331	201	2.22	1.47	3.14a	2.22
CP3	1.24	0.75	1.39ab	0.75	30.00	22.0a	32.00	22.60	262	183	303	192	2.16	1.08	3.08a	2.33
SE	0.03	0.03	0.02	0.02	2.10	0.89	0.61	1.42	14.80	7.92	16.70	8.53	0.13	0.15	0.08	0.17
Level of sig.	ns	ns	*	ns	ns	*	ns	ns	ns	ns	ns	ns	ns	ns	*	ns
**Residue**																
HR	1.28a	0.70	1.47a	0.80	30.87a	22.63a	33.87a	24.28	287a	193	338a	205	2.20	1.35	3.12a	2.18
LR	1.18b	0.72	1.32b	0.74	27.59b	17.13b	30.48b	22.40	237b	185	292b	207	2.15	1.32	2.81b	2.42
SE	0.02	0.06	0.02	0.03	0.79	1.22	0.46	1.28	14.30	10.70	7.24	14.70	0.12	0.08	0.10	0.16
Level of sig.	*	ns	*	ns	*	*	*	ns	*	ns	*	ns	ns	ns	*	ns

Abbreviations: MT, minimum tillage; CT, Conventional tillage; HR, High residue; NR, No residue; CP1- Wheat—T. Aus—T. Aman rice, CP2- Lentil—T. Aus—T. Aman rice and CP3-Mustard–Boro—T. Aman. Means separated by same letter were non-significant and different letter were significant at 5% level, ns, non-significant

*- significant at 5% level, SE = Standard error.

**Table 2:** Effects of management practices on soil organic carbon (SOC), basal respiration (BR), permanganate oxidizable carbon (POXC), and particulate organic carbon (POC) at calcareous site.

Factors	SOC (%)				BR (mg CO_2_/kg/d)				POC (g/kg)				POXC (mg/kg)			
	2018		2020		2018		2020		2018		2020		2018		2020	
**Tillage system**	**0–7.5 cm**	**7.5–15 cm**	**0–7.5 cm**	**7.5–15 cm**	**0–7.5 cm**	**7.5–15 cm**	**0–7.5 cm**	**7.5–15 cm**	**0–7.5 cm**	**7.5–15 cm**	**0–7.5 cm**	**7.5–15 cm**	**0–7.5 cm**	**7.5–15 cm**	**0–7.5 cm**	**7.5–15 cm**
ST	1.11b	1.03	1.28b	0.98	36.90a	14.70	38.65b	24.70	1.87	1.32	2.81b	2.40	240.80	164.85	285.80b	209.26
NT	1.21a	0.99	1.35a	1.10	28.95b	13.20	31.45c	16.95	1.95	1.45	3.20a	2.58	254.57	178.01	323.26a	211.10
CT	1.06b	0.96	1.20c	0.97	39.97a	16.72	43.72a	19.22	1.76	1.34	2.70b	2.22	222.50	168.85	265.00b	193.85
SE	0.02	0.02	0.02	0.07	1.99	1.61	1.30	4.57	0.11	0.08	0.05	0.23	8.43	4.72	9.57	9.66
Level of sig.	*	ns	*	ns	*	ns	*	ns	ns	ns	*	ns	ns	ns	*	ns
**Residue**																
HR	1.16a	0.98	1.34a	1.01	35.10	15.25	39.18	17.75	1.65	1.38	2.97	2.37	244.61	175.37	290.15	207.03
LR	1.09b	1.01	1.22b	1.02	35.45	14.50	36.70	22.83	2.08	1.36	2.83	2.43	233.97	165.78	290.56	202.44
SE	0.02	0.02	0.02	0.05	2.64	1.30	1.88	4.01	0.16	0.07	0.05	0.17	4.14	5.65	4.20	8.36
Level of sig.	*	ns	*	ns	ns	ns	ns	ns	ns	ns	ns	ns	ns	ns	ns	ns

Abbreviations:ST, Strip tillage; NT, No-tillage; CT, Conventional tillage; HR, High residue; LR, Low residue Means separated by same letter were non-significant and different letter were significant at 5% level; ns, non-significant; SE = Standard error.

Likewise, the ANOVA indicated that tillage, cropping system, and residue management had no effect on bulk density (ρb) in both soil depths under the non-calcareous site (Table 4). While average ρb values were similar in the MT (1.25 g/cm^3^) and CT (1.24 g/cm^3^) at 0–7.5 cm depths, it was 1.41 g/cm^3^ and 1.39 g/cm^3^, respectively, in the MT and CT systems in 2020. By contrast, ρb of the NT was statistically higher than the ST and CT at 0–7.5 cm depth in both years at the calcareous site (Table 6).

### Management systems’ impact on permanganate oxidizable, particulate organic, and total organic carbon pools

Tillage and residue retention had significant effects on total organic carbon (SOC) content at 0–7.5 cm depth in both years at the non-calcareous site. The MT had higher SOC (16.8% and 18.7% in 2018 and 2020, respectively) at 0–7.5cm depth than in the CT. When considering the impact of cropping systems, a significantly higher SOC content was observed in the LRR system than in the WRR and MRR systems in 2020. Regarding residue retention, SOC was significantly higher in the HR than in the LR, showing mean values of 1.28% and 1.18% in 2018 and 1.47% and 1.32% in 2020, respectively, at the surface soil. At the 7.5–15 cm depth, SOC was statistically similar in all tillage and cropping systems in both years (Table 1). At the calcareous site, tillage and residue retention had significant effects on SOC content at 0–7.5 cm depth in both years. The NT had a higher SOC than that of the ST and CT systems. The mean SOC was 1.21, 1.11, and 1.06% under the NT, ST, and CT, respectively, in 2018 and 1.35, 1.28, and 1.20% under the NT, ST, and CT, respectively, in 2020. With regard to residue retention, SOC was significantly higher in the HR than in the LR in both years. The SOC content decreased with soil depth (Table 2).

At the non-calcareous site, POXC was significantly higher in the MT than in the CT in 2020. Soils under the MT had a higher POXC by 5.7% and 27.3% than in the CT in 2018 and 2020, respectively, at 0–7.5 cm depth. Likewise, residue retention had a significant effect on POXC content at 0–7.5 cm depth in both years (Table 1). In contrast, tillage had significant effects on POXC at 0–7.5 cm in 2020 at the calcareous site. However, an opposite effect was observed on POXC in 2018. The NT and ST had 14.4% and 8.2% higher POXC than in the CT, respectively, in 2018 and 22% and 7.8% higher in the NT and ST than in the CT, respectively, in 2020. With regard to residue retention, the HR and LR were similar in both years. The POXC content was non-significant in all tillage and residue retention systems at the 7.5–15 cm depth in both years (Table 2).

At the non-calcareous site, the POC was significantly affected by tillage system at 0–7.5 cm depth in both years. Soils under the MT had 18.6% and 32.1% higher POC than under the CT in 2018 and 2020, respectively. Cropping system and residue retention had a significant effect on POC content at 0–7.5 cm depth in 2020, while these effects were opposite for 0–7.5 cm depth in both years. A higher POC content was found under the LRR and MRR systems than under the WRR cropping system at 0–7.5 cm depth in 2020 (Table 1). At the calcareous site, tillage and residue retention had non-significant effects on POC in 2018, while the NT had a significantly higher POC content than in the ST and CT in 2020. About 10.8% and 6.2% higher POC was observed in the NT and ST than in the CT in 2018. Similarly, about 18.5% and 4.1% higher POC was found in the NT and ST than in the CT in 2020. The HR had a higher content of POC than in the LR in both years (Table 2).

### Management systems’ impact on total soil organic carbon lability and management indices

At the non-calcareous site, the CMI based on POXC, POC, and MBC was significantly influenced by tillage systems at 0–7.5 cm depth in 2020, while the residue retention statistically influenced the CMI in both years at the same depth (Table 3). Higher values of CMI were observed in the MT than in the CT (Table 3). Soil carbon pool index (CPI), as an indicator of TOC accumulation, varied significantly in response to tillage and residue treatments at 0–7.5 cm soil depth in both years. The CPI in the MT increased by 18.5 and 18.8%, respectively, in 2018 and 2020 (Table 3). While the carbon lability index (CLI) of both POXC and POC was not significantly influenced by tillage, cropping system, and residue treatments in both years, in contrast, the CLI of MBC was significantly influenced by tillage at 0–7.5 cm depth in 2018 (Table 4).

**Table 3:** Effects of management practices on carbon pool index (CPI) and carbon management index (CMI) of particulate organic carbon (POC), permanganate oxidizable carbon (POXC), and microbial biomass carbon (MBC) at non-calcareous site.

Factors	CPI				CMI											
					POC				POXC				MBC			
	2018		2020		2018		2020		2018		2020		2018		2020	
**Tillage system**	**0–7.5 cm**	**7.5–15 cm**	**0–7.5 cm**	**7.5–15 cm**	**0–7.5 cm**	**7.5–15 cm**	**0–7.5 cm**	**7.5–15 cm**	**0–7.5 cm**	**7.5–15 cm**	**0–7.5 cm**	**7.5–15 cm**	**0–7.5 cm**	**7.5–15 cm**	**0–7.5 cm**	**7.5–15 cm**
MT	1.15a	0.94	1.15a	0.94	1.19a	2.15	1.32a	1.71	1.38	1.25	1.52a	1.16	1.77a	2.56	3.11a	1.45
CT	0.97b	1.00	0.97b	1.00	1.01b	1.40	0.97b	1.56	1.31	1.16	1.19b	1.06	1.16b	2.10	2.58b	1.14
SE	0.01	0.06	0.01	0.06	0.02	0.63	0.03	0.15	0.03	0.06	0.05	0.06	0.03	0.56	0.07	0.12
Level of sig.	*	ns	*	ns	*	ns	*	ns	ns	ns	*	ns	*	ns	*	ns
**Cropping system**																
CP1	1.03	0.96	1.03	0.96	1.07	2.23	1.01b	1.66	1.30	1.16	1.34	1.21	1.38b	1.94	2.57b	1.27
CP2	1.09	0.93	1.09	0.93	1.13	1.96	1.21a	1.45	1.39	1.28	1.42	1.09	1.57a	3.01	3.36a	1.34
CP3	1.07	1.02	1.07	1.02	1.10	1.14	1.21a	1.78	1.34	1.17	1.30	1.04	1.45ab	2.04	2.61b	1.27
SE	0.02	0.03	0.02	0.03	0.07	0.41	0.04	0.26	0.08	0.05	0.07	0.04	0.04	0.51	0.19	0.23
Level of sig.	ns	ns	ns	ns	ns	ns	*	ns	ns	ns	ns	ns	*	ns	*	ns
**Residue**																
HR	1.10a	0.97	1.10a	0.97	1.10	1.94	1.21	1.58	1.47	1.23	1.45a	1.13	1.55a	2.50	3.22a	1.41
LR	1.02b	0.97	1.02b	0.97	1.09	1.61	1.08	1.68	1.22	1.18	1.25b	1.10	1.39b	2.16	2.47b	1.18
SE	0.02	0.07	0.02	0.07	0.07	0.18	0.04	0.27	0.07	0.07	0.03	0.08	0.04	0.79	0.13	0.14
Level of sig.	*	ns	*	ns	ns	ns	ns	ns	*	ns	*	ns	*	ns	*	ns

Abbreviations: MT, minimum tillage; CT, Conventional tillage; HR, High residue; NR, No residue; CP1- Wheat—T. Aus—T. Aman rice, CP2- Lentil—T. Aus—T. Aman rice and CP3-Mustard–Boro—T. Aman. Means separated by same letter were non-significant and different letter were significant at 5% level, ns, non-significant

*- significant at 5% level, SE = Standard error.

**Table 4:** Effects of management practices on soil bulk density (ρb) and carbon lability index (CLI) of particulate organic carbon (POC), permanganate oxidizable carbon (POXC), and microbial biomass carbon (MBC) at non-calcareous site.

Factors	ρb (g/cm^3^)				CLI											
					POC				POXC				MBC			
	2018		2020		2018		2020		2018		2020		2018		2020	
**Tillage system**	**0–7.5 cm**	**7.5–15 cm**	**0–7.5 cm**	**7.5–15 cm**	**0–7.5 cm**	**7.5–15 cm**	**0–7.5 cm**	**7.5–15 cm**	**0–7.5 cm**	**7.5–15 cm**	**0–7.5 cm**	**7.5–15 cm**	**0–7.5 cm**	**7.5–15 cm**	**0–7.5 cm**	**7.5–15 cm**
MT	1.23	1.38	1.25	1.41	1.05	2.19	1.09	2.08	1.21	1.54	1.27	1.34	1.56a	3.08	2.61	0.76
CT	1.22	1.33	1.24	1.39	1.04	1.53	0.96	1.83	1.35	1.34	1.17	1.20	1.21b	3.07	2.53	0.85
SE	0.04	0.01	0.03	0.03	0.01	0.66	0.03	0.12	0.03	0.18	0.05	0.07	0.04	0.67	0.03	0.02
Level of sig.	ns	ns	ns	ns	ns	ns	ns	ns	ns	ns	ns	ns	*	ns	ns	ns
**Cropping system**																
CP1	1.22	1.37	1.23	1.40	1.04	2.25	0.96	2.01	1.26	1.40	1.27	1.37	1.34	2.31	2.41	0.83
CP2	1.23	1.36	1.24	1.41	1.05	2.07	1.03	1.62	1.31	1.56	1.22	1.21	1.45	4.21	2.92	0.95
CP3	1.25	1.35	1.25	1.38	1.03	1.26	1.08	2.24	1.27	1.36	1.18	1.23	1.34	2.71	2.37	0.63
SE	0.03	0.03	0.04	0.03	0.07	0.39	0.04	0.24	0.09	0.15	0.06	0.04	0.06	1.05	0.15	0.15
Level of sig.	ns	ns	ns	ns	ns	ns	ns	ns	ns	ns	ns	ns	ns	ns	ns	ns
**Residue**																
HR	1.22	1.36	1.24	1.38	1.01	2.07	1.02	1.92	1.35	1.50	1.24	1.26	1.39	3.23	2.78	0.75
LR	1.24	1.37	1.25	1.42	1.08	1.64	1.03	1.99	1.21	1.38	1.20	1.28	1.36	2.92	2.36	0.86
SE	0.03	0.03	0.02	0.02	0.08	0.27	0.04	0.24	0.06	0.16	0.03	0.07	0.05	0.82	0.14	0.12
Level of sig.	ns	ns	ns	ns	ns	ns	ns	ns	ns	ns	ns	ns	ns	ns	ns	ns

Abbreviations: MT, minimum tillage; CT, Conventional tillage; HR, High residue; NR, No residue; CP1- Wheat—T. Aus—T. Aman rice, CP2- Lentil—T. Aus—T. Aman rice and CP3-Mustard–Boro—T. Aman. Means separated by same letter were non-significant and different letter were significant at 5% level, ns, non-significant

*- significant at 5% level, SE = Standard error.

At the calcareous site, the CMI based on POC, POXC, and MBC was statistically similar between the residue treatments. The NT had higher CMI values calculated based on POC, POXC, and MBC than in the ST and CT at 0–7.5 cm depth in 2020 (Table 5). Likewise, the CPI under the NT significantly increased when compared to the ST and CT in both years. Considering the residue retention of CPI value and CMI of POXC and MBC, the HR was statistically higher than NR in both years at 0–7.5 cm soil depth (Table 5). The CLI based on POC, POXC, and MBC was not influenced by tillage and residue treatments between depths and years (Table 6).

**Table 5:** Effects of management practices on carbon pool index (CPI) and carbon management index (CMI) of particulate organic carbon (POC), permanganate oxidizable carbon (POXC), and microbial biomass carbon (MBC) at calcareous site.

Factors	CPI				CMI											
					POC				POXC				MBC			
	2018		2020		2018		2020		2018		2020		2018		2020	
**Tillage system**	**0–7.5 cm**	**7.5–15 cm**	**0–7.5 cm**	**7.5–15 cm**	**0–7.5 cm**	**7.5–15 cm**	**0–7.5 cm**	**7.5–15 cm**	**0–7.5 cm**	**7.5–15 cm**	**0–7.5 cm**	**7.5–15 cm**	**0–7.5 cm**	**7.5–15 cm**	**0–7.5 cm**	**7.5–15 cm**
ST	1.05b	1.09	1.15ab	1.03	1.13	1.20	1.25b	2.06	1.12	1.08	1.04ab	1.20	1.83ab	1.34	1.48ab	1.67
NT	1.14a	1.05	1.22a	1.15	1.17	1.31	1.47a	1.69	1.17	1.17	1.18a	1.22	2.10a	1.47	1.94a	1.63
CT	1.01b	1.01	1.08b	1.02	1.05	1.19	1.21b	1.47	1.02	1.09	0.96b	1.09	1.10b	1.20	1.20b	1.31
SE	0.02	0.02	0.02	0.07	0.08	0.09	0.04	0.29	0.04	0.03	0.03	0.06	0.19	0.28	0.13	0.22
Level of sig.	*	ns	*	ns	ns	ns	*	ns	ns	ns	*	ns	*	ns	*	ns
**Residue**																
HR	1.10a	1.04	1.21a	1.06	0.97	1.24	1.34	1.89	1.13	1.15	1.06	1.20	1.68	1.41	1.62	1.59
LR	1.03b	1.06	1.09b	1.07	1.26	1.22	1.29	1.59	1.08	1.08	1.06	1.14	1.66	1.27	1.46	1.48
SE	0.02	0.02	0.02	0.05	0.11	0.07	0.03	0.26	0.02	0.04	0.02	0.04	0.22	0.20	0.06	0.21
Level of sig.	*	ns	*	ns	ns	ns	ns	ns	ns	ns	ns	ns	ns	ns	ns	ns

Abbreviations:ST, Strip tillage; NT, No-tillage; CT, Conventional tillage; HR, High residue; LR, Low residue Means separated by same letter were non-significant and different letter were significant at 5% level; ns, non-significant; SE = Standard error.

**Table 6:** Effects of management practices on soil bulk density (ρb) and carbon lability index (CLI) of particulate organic carbon (POC), permanganate oxidizable carbon (POXC), and microbial biomass carbon (MBC) at calcareous site.

Factors	ρb (g/cm^3^)				CLI											
					POC				POXC				MBC			
	2018		2020		2018		2020		2018		2020		2018		2020	
**Tillage system**	**0–7.5 cm**	**7.5–15 cm**	**0–7.5 cm**	**7.5–15 cm**	**0–7.5 cm**	**7.5–15 cm**	**0–7.5 cm**	**7.5–15 cm**	**0–7.5 cm**	**7.5–15 cm**	**0–7.5 cm**	**7.5–15 cm**	**0–7.5 cm**	**7.5–15 cm**	**0–7.5 cm**	**7.5–15 cm**
ST	1.22ab	1.38	1.25b	1.42	1.09	1.08	1.09	2.73	1.06	0.99	0.91	1.24	1.74	1.30	1.30	1.68
NT	1.29a	1.40	1.33a	1.41	1.01	1.25	1.20	1.48	1.02	1.11	0.97	1.07	1.82	1.42	1.60	1.47
CT	1.157b	1.36	1.19b	1.43	1.04	1.15	1.13	1.43	1.02	1.07	0.89	1.09	1.09	1.19	1.10	1.27
SE	0.02	0.01	0.02	0.02	0.09	0.08	0.04	0.78	0.03	0.03	0.03	0.11	0.18	0.31	0.11	0.21
Level of sig.	*	ns	*	ns	ns	ns	ns	ns	ns	ns	ns	ns	ns	ns	ns	ns
**Residue**																
HR	1.21	1.38	1.23b	1.40	0.88	1.17	1.10	2.31	1.03	1.10	0.87	1.19	1.52	1.38	1.34	1.54
LR	1.24	1.39	1.29a	1.43	1.22	1.15	1.17	1.45	1.05	1.02	0.97	1.09	1.58	1.23	1.32	1.41
SE	0.02	0.01	0.01	0.01	0.10	0.06	0.06	0.64	0.03	0.03	0.01	0.09	0.19	0.18	0.06	0.22
Level of sig.	ns	ns	*	ns	ns	ns	ns	ns	ns	ns	ns	ns	ns	ns	ns	ns

Abbreviations:ST, Strip tillage; NT, No-tillage; CT, Conventional tillage; HR, High residue; LR, Low residue Means separated by same letter were non-significant and different letter were significant at 5% level; ns, non-significant; SE = Standard error.

### Management systems’ impact on total soil organic carbon stocks

At the non-calcareous site, SOC stock was not influenced by tillage, cropping system, and residue treatments (Figs 1D, 2D and 3D) being higher in the MT than in the CT treatments at both depths in 2018. In contrast, the SOC stocks, in response to tillage and residue treatments, were significantly higher at 0–7.5 cm depth in 2020. While the SOC stocks in the MT and CT was 14.2 and 11.92 Mg ha^−1^, respectively, at 0–7.5 cm, it was 8.4 and 8 Mg ha^−1^, respectively, at 7.5–15 cm depth in 2020. Likewise, the POC stock was significantly influenced by tillage treatments at 0–7.5 cm depth in both years. The POC stocks in response to cropping systems were statistically similar in both years, while the residue treatment had a significant effect at 0–7.5 cm depth in 2020 (Figs 1C, 2C and 3C). The POXC stocks, in contrast, were significantly influenced among the tillage treatments in 2020 at 0–7.5 cm depth, while it was identical in cropping system and residue treatments (Figs 1A, 2A and 3A). The higher POXC stock was observed in the MT than in the CT. As expected, the SOC, POC, POXC, and MBC stocks were declined at 7.5–15 cm when compared to 0–7.5 cm depth.

At the calcareous site, tillage had significant impacts on SOC stocks at 0–7.5 cm depth in both years, while the residue had a significant effect at 0–7.5 cm depth only in 2020 (Figs 4D and 5D). The POC stock was significantly influenced by tillage at 0–7.5 cm depth in 2020, while the residue treatments had a similar effect at all soil depths in both years. The higher POC stock was observed in the NT than in the ST and CT at 0–7.5 cm depth in 2020. In contrast, the POXC and MBC stocks were significantly influenced by tillage treatments at 0–7.5 cm depth in both years, whereas higher values of POXC and POC were observed in the NT than in the ST and CT in both years. Considering crop residue management, both POXC and MBC were statistically similar, being higher in the HR than in the LR treatments at all depths in both years (Figs 4 and 5).

### Relationship among total soil organic carbon pools

**Fig 6:**
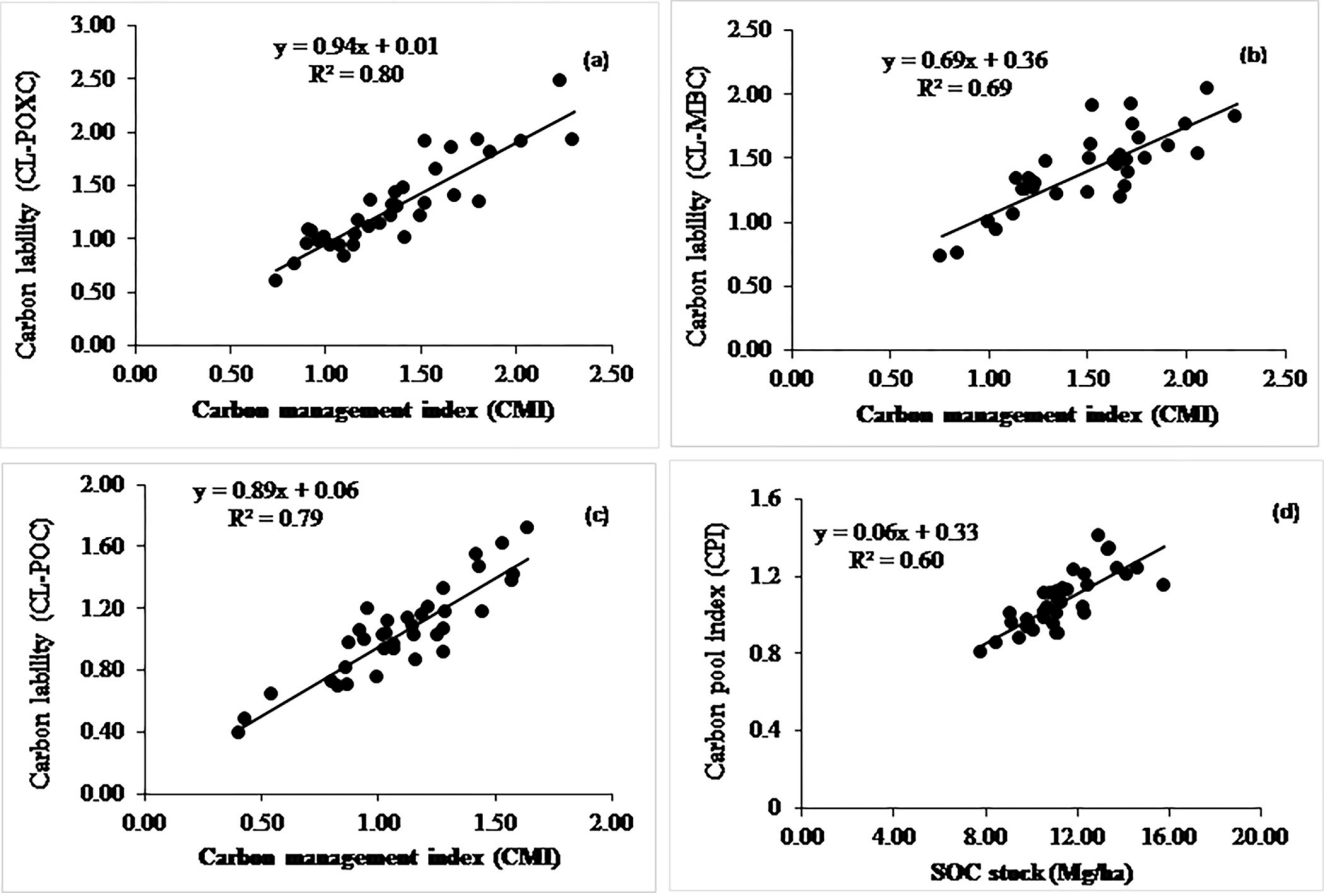
Relationship between (a) CMI and CLI of POXC, (b) CMI and CLI of MBC, (c) CMI and CLI of POC, and (d) CPI with SOC stocks at non-calcareous site.

**Fig 7:**
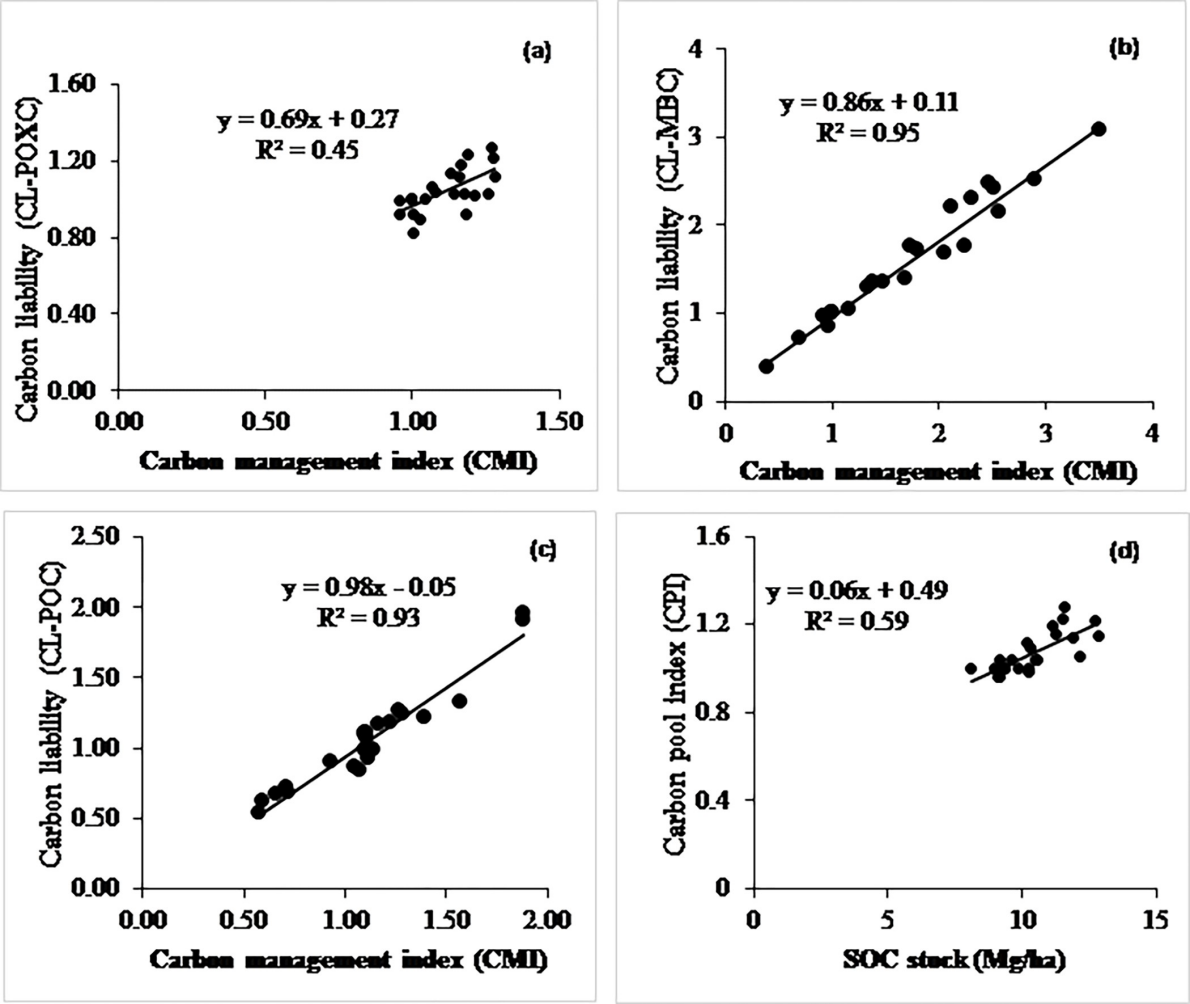
Relationship between (a) CMI and CLI of POXC, (b) CMI and CLI of MBC, (c) CMI and CLI of POC, and (d) CPI with SOC stock at calcareous site.

At the non-calcareous site, the CMI positively and linearly correlated with the carbon lability of POXC, POC, and MBC (Fig 6). The SOC stock linearly accounted for 60% (R^2^ = 0.6) of the variability in the CPI (Fig 6D). Likewise, the CMI significantly accounted for 80, 69, and 79% variability in the POXC, MBC, and POC pools, respectively, (Fig 6A–6C). At the calcareous site, the CMI linearly correlated with the carbon liability of POXC, POC, and MBC (Fig 7). Likewise, the CMI significantly accounted for 45, 95, and 93% variability in the POXC, MBC, and POC, respectively, (Fig 7A–7C). Moreover. the SOC stock linearly accounted for 60% of the variability in the CPI (Fig 7D).

## Discussions

### Management impacts on soil microbial biomass and associated biological properties

Soil MBC, as a labile carbon, is vital for catalyzing C and N transformations, regulating nutrient cycling, and maintaining soil health [2]. Our results suggested that MBC was consistently influenced by the variable effects of tillage, residue, and cropping system as part of the CA on its activity. Undisturbed soils in CA system may have harbored a more diversified microbial community. The MBC content under the NT and RT (ridge tillage) was significantly higher than the MP (mold-board plough) at 0–5 cm depth due to surface accumulation of crop residues with a greater biodiversity under undisturbed site conditions [32]. A significantly higher MBC may have acted positively to affect a higher level of active or POXC to support efficient biological activities [33]. The amount of residual C and nutrients returning to the undisturbed surface soil has a great impact on variable shift in the biologically labile pools of SOC and MBC diversity. A significant increase in the MBC and MBC:SOC were due to an increase in the size of MBC pool related to greater availability of active C [7]. With an increase or depletion in SMBC, there was a pronounced effect on the active C or vice-versa [29]. Crop rotation, as an integral component of CA, had a positive effect on the MBC, active C, and SOC lability, and crop rotations to support crop residue C diversity, maintain rhizosphere effects, and exert synergistic effects on the MBC and associated biological activities [9, 34].

Higher BR rates under the CT than under the MT soil were most likely associated with greater catabolism of labile C due to accelerated aeration and its consequent effect on SOC depletion in response to annual plowing. A 14% decrease was reported in the cumulative CO_2_ flux from soils under the NT when compared to the CT [35]. Higher CO_2_ fluxes from the CT is not surprising, because tillage practices disrupt macroaggregates, which exposes labile C to accelerate oxidation of SOM, thus increasing the emission of CO_2_. Due to a lack of disturbance in CA soils and the stabilization of C in microaggregates (CA favor soil aggregate formation and stability) that are sealed in macroaggregates, the NT has been found to reduce BR rates [36]. Basal respiration has been suggested as a sensitive antecedent indicator of SOC dynamics. Crop rotation, when combined with the NT, is a biological diversity management option to develop soil functionality [9]. Several studies have reported that microbes are allocating more C energy for cell growth with a significant decrease in C loss to carry out soil quality functions [37].

### Management systems impact on total organic, permanganate oxidizable and particulate organic carbon pools

Significant changes in SOC contents in response to variable tillage practices, especially under the CT and MT systems, were due to plowing disturbance on SOM decomposition. It is reported that CA such as zero tillage (ZT) can influence the distribution of SOC with higher SOC content in surface layers than with the CT due to the limited biodiversity and size of microbial communities in soils under the CT [38]. The CT disrupted macroaggregates, which reduces the physical protection of SOC, especially labile C pool. That is why under the NT, macroaggregate turnover rate is slower than the CT, which indicates a higher protection level of SOC. Intensive tillage, such as CT results in SOC loss, thereby increases greenhouse gas emissions with an associated decrease in SOC content [36].

Several studies have reported that greater addition of crop residues (HR) under the cropping systems and ZT significantly increased SOC content in the 0–25 cm depth when compared to the effects of deep tillage [39]. A slower decomposition of crop residues under the ZT with higher metabolic efficiency (anabolism) of microbes caused an accumulation of SOC under CA-based managements. The introduction of the NT system with an increase in crop residues results in an increase in the SOC [40]. It is reported that rice straw retention was effective for increasing SOC content and, consequently, improving soil fertility and productivity in clayey soils [41]. Crop residue returning is recommended as an important management practice for improving soil quality and crop yield [42] and, thus, has been globally promoted. It is reported that crop residue returning has a strong effect on SOC dynamics [43] and greenhouse gas emissions [44]. Frequent tillage along with residue removal caused depletion in SOC content, specifically from the top 0–15 cm depths. It has been estimated that loss of SOC stock in CT soils was as high as 75% of the SOC stock in the native lands [45]. Several studies reported that addition of organic materials such as crop residues significantly increased the SOC content by 29.6 to 119.8% and 10.3 to 36.3% at 0–15 and 15–30 cm, respectively, when compared to N fertilizer application by farmers. Organic amendments also improved soil labile carbon fractions, especially the MBC and POC [10].

A significantly higher SOC content observed in the legume-based cropping system (LRR) compared to the rice-based systems was due to the effects of N recycling from leguminous crop residues. Lentil stover C:N is lower than rice and wheat, which has higher microbial preference for decomposition, suggesting higher microbial efficiency and growth. This eventually increases MBC in soil as a part of SOC. It is also reported that continuous mono-cropping led to rapid depletion of labile SOC pools [29]. Higher concentration of POXC and POC was found in legume-based cropping patterns. The SOC content was declined in deep tillage after each cropping cycle of wheat—mungbean—T. aman rice, whereas SOC gradually accumulated due to lack of or minimum disturbance after the four cropping cycles [46]. Higher SOC content was found in a rice—wheat—legume rotation than in a rice—wheat—fallow system in Bangladesh [47]. A similar study reported that strip planting along with residue return under lentil—mung bean—rice and wheat -mung—rice rotations had positive effects on SOC pools after 1.5 yr, when compared with the CT and limited residue return in soil [9].

Our research showed that a higher level of SOC, POXC, and POC was found in the MT at the non-calcareous site and in the NT than in the CT at the calcareous site due to the effects of contrasting management practices. Over the years, research studies have shown that labile carbon fractions were stimulated by sustainable agricultural practices such as reduced tillage, cover cropping, and land use [48]. Labile SOC pool, such as POXC, is considered to be a source of food, energy, and a regulator of soil quality functions in response to management practices [28, 29, 49]. Residue decomposition byproducts and microbial biomass comprise the main source of labile organic matter in soil ecosystems [50]. A number of studies have indicated reduced tillage practices and the addition of crop residue increase the labile carbon pool [51].

Retention of crop residues and the addition of bio-inoculants help to maintain C and N balance in soil and enrich the labile C pool in rice—legume—rice cropping systems [52]. An efficient metabolism of the labile carbon is associated with higher SOC concentration in the ZT, when compared to the CT [53]. Previous research showed that higher crop residue retention produced a proportionally greater increase in labile carbon than in SOC, when compared to limited crop residue addition [54]. It is expected that the MT had a higher active C pool with a legume-based cropping system than with a cereal-based cropping system. Moreover, the passive C pool was also boosted by legume-based cropping sequences [55]. SOC content and its lability can be increased by replacing the CT or mono-cropping systems with continuous NT and cropping diversity [56].

### Management impacts on soil carbon stocks and soil carbon lability and management indices

Increased SOC, POC, POXC, and MBC stocks at both the non-calcareous and calcareous sites were due to greater residue retention under a legume-based cropping pattern, which agrees with previous research studies [57]. Low temperatures, the acidic environment, and the partially anaerobic conditions are favorable for the slower decomposition of the organic substances that were composed of a series of fractions that vary in their turnover time, decomposition degree, and recalcitrance [58]. The SOC stocks in the upper layers of the soil are more sensitive and receptive to contrasting changes in land use and management practices. The conservation systems that reduce disturbance and retain residue at the soil surface can improve site conditions, especially porosity, aggregate stability, water infiltration, and nutrient availability. In a three-year study of a rice—wheat system, SOC content was 0.22% greater under the NT raised bed than under the CT [59]. Studies on the plains of Nepal reported 9.9% greater SOC in the 0–50 cm profile under the NT than under the CT in a rice—wheat system [60]. Under the NT, there was 28.3% higher SOC content at 0–5 cm depth of the NT than that of the CT system.

The CMI is a sensitive indicator of SOC dynamics associated with soil quality to evaluate the capacity of management systems that have close correlation with soil physical, chemical, and biological properties [61, 62]. In the present study, the effect of a legume-based cropping pattern contained the highest values of CMI of MBC, which agrees with previous research [61–63]. This is due to the effect of an increase in annual organic C input and the efficiency of microbial biomass, thus increasing the accumulation of labile C in SOC [21]. Similar results have reported that manure alone and manure with chemical fertilizers significantly increased CMI when compared with other chemical fertilizer treatments [63]. Inclusion of legumes and balanced nutrient management practices played an important role to accumulate SOC, carbon lability, and the CMI under the maize-based cropping system [64].

The highest values of CMI were observed in summer legumes such as cowpea and pigeon pea [61]. Soils with higher or lower values of CPI are considered as indicators of SOC accumulation or depletion [30]. Soils with higher CMI values are considered to be better managed with higher C-use efficiency towards improved soil quality [29, 61, 62]. Early changes in SOC lability and accumulation or depletion can be detected by calculating CPI and CMI values, which are undetectable when considering SOC values [29]. Soils with higher CMI values are considered to be better managed with higher C-use efficiency towards improved soil quality [9, 61].

## Conclusions

Reduced tillage with residue management improved SOC by reducing labile C turnover rates with greater microbial efficiency (anabolism). Undisturbed soils such as reduced tillage favor slower residue and SOC accumulation, resulting in higher labile C content of the soils in both medium and short-term evaluation phases of our study, which suggests to continue our experiment for a longer period of time to improve soil quality and crop productivity at both calcareous and non-calcareous sites. However, the concentration of SOC pools were higher in the non-calcareous sites than in the calcareous site, suggesting adoption of CA practices. Inclusion of legumes in the cropping pattern increased SOC content and its lability with efficient BR rates via microbial anabolism due to their lower C:N. A significant linear relationship between the CPI and SOC stock indicated a proportionally more SOC sequestration by conservation land management practices. Likewise, the linear relationship of CMI and C lability of POXC, POC, and MBC indicated the positive impact of CA managements on labile C accumulation. An early detection of temporal changes from labile SOC pools, CPI, and CMI in response to variable agricultural practices can be useful to evaluate soil quality, which is beneficial for sustainable agriculture by increasing SOC stocks in the intensive rice-based cropping systems in the south Asia.
